# Hematometabolic Index as a New Discriminator of Cardiometabolic Risk in Middle-Aged Men with Polycythemia and High Leukocyte Count in Peripheral Blood

**DOI:** 10.1089/met.2023.0011

**Published:** 2023-06-16

**Authors:** Ichiro Wakabayashi, Takashi Daimon

**Affiliations:** ^1^ Department of Environmental and Preventive Medicine, School of Medicine, Hyogo Medical University, Nishinomiya, Japan.; ^2^ Department of Biostatistics, School of Medicine, Hyogo Medical University, Nishinomiya, Japan.

**Keywords:** cardiovascular disease, hematometabolic index, leukocyte count, metabolic syndrome, polycythemia

## Abstract

**Background:**

Both polycythemia and high leukocyte count are associated with the risk of cardiovascular disease. However, it remains to be determined whether polycythemia and high leukocyte count show synergistic increasing effects on cardiometabolic risk.

**Methods:**

Cardiometabolic risk was evaluated by cardiometabolic index (CMI) and metabolic syndrome in a cohort of middle-aged men (*n* = 11,140) who underwent annual health check-up examinations. The subjects were divided into three tertile groups by hemoglobin concentration or leukocyte count in peripheral blood, and their relations with CMI and metabolic syndrome were investigated. A new index, named hematometabolic index (HMI), was defined as the product of hemoglobin concentration (g/dL)-minus-13.0 and leukocyte count (/μL)-minus-3000.

**Results:**

When the subjects were further classified by tertiles for hemoglobin concentration and leukocyte count into nine groups, the odds ratios for high CMI and metabolic syndrome of the group categorized in the highest (third) tertiles for both hemoglobin concentration and leukocyte count versus the group of the lowest (first) tertiles for both of them were highest among the nine groups. In receiver-operating characteristic (ROC) analysis for relationships of HMI with high CMI and metabolic syndrome, areas under the ROC curves (AUCs) were significantly larger than the reference level and tended to be smaller with an increase in age. In subjects from 30 to 39 years of age, the AUC for the relationship between HMI and metabolic syndrome was 0.707 (0.663–0.751) and the cutoff of HMI was 9850.

**Conclusions:**

HMI, reflecting hemoglobin concentration and leukocyte count, is thought to be a possible marker for discriminating cardiometabolic risk.

## Introduction

Atherosclerosis, the basic pathogenesis of cardiovascular diseases, is interpreted as a state of chronic low-grade inflammation in arterial walls.^1,2^ Therefore, it is plausible that a sustained increase in circulatory leukocytes and acute-phase reactants is a risk factor for atherosclerotic diseases, including ischemic heart disease, stroke, and peripheral arterial disease.^3,4^ In addition, hypercholesterolemia, a major risk factor for cardiovascular diseases, has been shown to induce proliferation of hematopoietic stem cell progenitors,^5^ resulting in an increase in leukocyte count.

Polycythemia, defined as an abnormal elevation of hemoglobin and/or hematocrit in peripheral blood, is also a risk factor for cardiovascular disease^6,7^: polycythemia causes an elevation of blood viscosity, resulting in an increase in hemodynamic shear stress, which induces thrombotic cardiovascular events. Polycythemia is also associated with cardiometabolic risk factors, including obesity, hypertension, dyslipidemia, and diabetes.^8^ The threshold levels of hemoglobin and hematocrit for diagnosing polycythemia vera, the most common disease categorized in primary polycythemia, were lowered in the new criteria by WHO in 2016.^9^ The number of individuals with polycythemia is therefore expected to increase considerably. Primary polycythemia is a rare disease,^10,11^ and most cases of polycythemia in the general population are cases of secondary polycythemia, which is due to an increase in erythropoietin and is caused by various conditions, including smoking, sleep apnea, high altitude, chronic cardiopulmonary diseases, and erythropoietin-producing tumors.^12^ Moreover, sleep apnea is closely associated with obesity, a central cardiovascular risk factor.^13^

Although individuals with polycythemia or high leukocyte count in peripheral blood have a higher risk for cardiovascular events, it remains to be determined whether polycythemia and high leukocyte count show synergistic increasing effects on cardiovascular risk. If so, an index reflecting both erythrocyte- and leukocyte-related variables may be useful for evaluating cardiovascular risk since examinations of blood cell counts and erythrocyte-related variables, such as hemoglobin concentration and hematocrit, are usually included in general health check-up examinations.

In this study, we, therefore, investigated the relationships of polycythemia alone and high leukocyte count alone and both of them with states of plural cardiovascular risk factors, such as high cardiometabolic index (CMI) and metabolic syndrome. The former reflects the two risk factors, including dyslipidemia and visceral obesity,^14^ and the latter reflects the four risk factors, including dyslipidemia, visceral obesity, hypertension, and diabetes.^15^ We proposed a new index, named hematometabolic index (HMI) that is calculated as the product of leukocyte count and hemoglobin concentration after modification, and we investigated the relationship between HMI and cardiometabolic risk.

## Methods

### Subjects

The subjects were Japanese men 30–65 years of age (*n* = 11,140) who had received periodic health checkup examinations at workplaces in Yamagata Prefecture in Japan. This study was approved by the Ethics Committee of Yamagata University School of Medicine (No. 112 from April 2005 to March 2006, approved on March 13, 2006) and the Hyogo College of Medicine Ethics Committee (No. 3003 in 2020). Those showing hemoglobin levels of <12 g/dL (*n* = 84) and/or showing leukocyte count of ≥12,000/μL (*n* = 121) were excluded from the subjects of this study. The same original database of subjects was used in this study and our former study,^8^ but the subjects for analysis were slightly different in those studies because of a difference in the exclusion criteria.

Histories of cigarette smoking, alcohol consumption, regular exercise, illness, and therapy for illness were surveyed by questionnaires. In the self-written questionnaire paper, participants were first asked “Are you a habitual cigarette smoker?” Cigarette smokers were defined as participants who had smoked for 6 months or longer and had smoked for the past month or longer. Then the participants who were smokers were further asked “What is your average cigarette consumption per day?” The response categories for this question were “20 or less cigarettes per day,” “21 or more and 40 or less cigarettes per day,” and “41 or more cigarettes per day.” Because the percentage of subjects with very heavy cigarette consumption (41 or more cigarettes per day) was very low [0.5% (*n* = 55)], the subjects were divided into three groups of nonsmokers, light smokers (20 or less cigarettes per day) and heavy smokers (21 or more cigarettes per day) in this study.

Average alcohol consumption of each subject per week was also reported on questionnaires. The frequency of habitual alcohol drinking was asked in the questionnaire as “How frequently do you drink alcohol?” The frequency of weekly alcohol drinking was categorized as “every day” (regular drinkers), “sometimes” (occasional drinkers), and “never” (nondrinkers). Subjects with a habit of regular exercise were defined as those doing exercise almost every day for 30 min or longer per day.

### Measurements

Height and body weight were measured with light clothes at the health checkup. Body mass index was calculated as weight in kilograms divided by the square of height in meters. Waist circumference was measured at the navel level according to the recommendation of the definition of the Japanese Committee for the Diagnostic Criteria of Metabolic Syndrome.^16^ Blood pressure was measured by trained nurses, who were part of the local health checkup company, with a mercury sphygmomanometer once on the day of the health checkup after each subject had rested quietly in a sitting position. Korotkoff phase V was used to define diastolic pressure.

Fasted blood was collected from each subject in the morning, and a part of the blood was immediately transferred to a 2-mL glass tube containing 3.8 mg EDTA-2K. Hemoglobin concentration was measured by the sodium lauryl sulfate hemoglobin method and leukocyte count was measured by flow cytometry (with a red laser at 633 nm) using an automatic hematology analyzer (Sysmex XE-2100; Sysmex Corp., Kobe, Japan). A new index, named HMI, was defined as the product of hemoglobin concentration (g/dL)-minus-13.0 and leukocyte count (/μL)-minus-3000. Subjects showing a low hemoglobin concentration (13 g/dL or lower) and a low leukocyte count (3000/μL or lower) were excluded in receiver-operating characteristic (ROC) analysis because their HMI values were zero or lower.

Serum triglycerides and high-density lipoprotein (HDL) cholesterol were measured by enzymatic methods using commercial kits, Pureauto S TG-N and Cholestest N-HDL (Sekisui Medical Co., Ltd., Tokyo, Japan), respectively. Hemoglobin A_1c_ was measured by the NGSP (National Glycohemoglobin Standardization Program)-approved technique using the latex cohesion method with a commercial kit (Determiner HbA_1c_, Kyowa Medex, Tokyo, Japan). Since the standards of hemoglobin A_1c_ used for measurement are different in the NGSP method and JDS (the Japan Diabetes Society) method, hemoglobin A_1c_ values were calibrated by using a formula proposed by JDS^17^: hemoglobin A_1c_ (NGSP) (%) = 1.02 × hemoglobin A_1c_ (JDS) (%)+0.25 (%). CMI was defined as the product of two ratios: the ratio of waist circumference (cm) to height (cm) and the ratio of triglycerides (mg/dL) to HDL cholesterol (mg/dL).^14^ High CMI was defined as 1.748 or higher.^14^

Metabolic syndrome was defined, according to the criteria by the International Diabetes Federation^15^ with a slight modification, as the presence of two or more risk factors in addition to visceral obesity diagnosed as high waist-to-height ratio. Risk factors included in the criteria are visceral obesity (high waist-to-height ratio), high blood pressure, dyslipidemia (low HDL cholesterol and/or high triglycerides) and hyperglycemia evaluated by hemoglobin A_1c_. The criterion for each risk factor in metabolic syndrome was defined as follows: visceral obesity, waist-to-height ratio ≥0.5^18^; high blood pressure, systolic blood pressure ≥140 mmHg and/or diastolic blood pressure ≥90 mmHg^19^; low HDL cholesterol, HDL cholesterol <40 mg/dL; high triglycerides, triglycerides ≥150 mg/dL^20,21^; hyperglycemia, hemoglobin A_1c_ ≥6.5%.^22^ Subjects receiving drug therapy for hypertension and diabetes were also included in the above definitions of high blood pressure and hyperglycemia, respectively.

### Statistical analyses

Continuous variables are summarized as means with standard deviations or medians with interquartile ranges, as appropriate. Categorical variables are summarized as frequencies and percentages. Dichotomous variables were compared by using multiple logistic regression analysis, in which the crude and adjusted odds ratios were estimated with their corresponding 95% confidence intervals. The multivariable analyses included adjustments for age, habits of smoking, alcohol drinking and regular exercise, and a history of medication therapy for dyslipidemia. ROC analysis was performed to examine an optimal cutoff point of HMI. The optimal cutoff point was selected by minimizing the distance to the top-left corner of the ROC curve for each cutoff point. The area under the ROC curve (AUC) and 95% confidence interval were estimated empirically.

All probability (*P*) values are two-sided. Statistical significance was defined when a *P* value was <0.05. A computer software program (IBM SPSS Statistics for Windows, Version 25.0; IBM Corp., Armonk, NY, USA) and R (Version 4.0.3. Vienna, Austria: https://www.R-project.org/) were used for the statistical analyses.

## Results

### Characteristics of the subjects

Table 1 shows characteristics of the subjects. The percentages of smokers, alcohol drinkers, and subjects with a habit of regular exercise were 55.8%, 79.7%, and 11.7%, respectively. The percentages of the subjects having abdominal obesity (high waist-to-height ratio), hypertension, dyslipidemia, and diabetes were 41.0%, 29.2%, 34.2%, and 6.2%, respectively. The percentages of the subjects with high CMI and metabolic syndrome were 22.7% and 11.2%, respectively. The cutoff values for the second and third tertiles for hemoglobin were 14.8 and 15.6 g/dL, respectively. The cutoff values for the second and third tertiles for leukocyte count were 5700/μL and 7100/μL, respectively.

**Table 1.** Characteristics of Overall Subjects

Variables	Values
Number	11,140
Age (years)	47.1 ± 9.0
Smokers (%)	55.8%
Drinkers (%)	79.7%
Regular exercise (%)	11.7%
Hemoglobin (g/dL)	15.17 ± 0.99
	First tertile: 14.10 ± 0.54
	Second tertile 15.16 ± 0.22
	Third tertile: 16.22 ± 0.58
Leukocyte count (/μL)	6495 ± 1648
	First tertile: 4809 ± 600
	Second tertile: 6319 ± 396
	Third tertile: 8407 ± 1110
Body mass index (kg/m^2^)	23.5 ± 3.3
Waist circumference (cm)	83.2 ± 9.0
Waist-to-height ratio	0.490 ± 0.053
High waist-to-height ratio (%)	41.0
Systolic blood pressure (mmHg)	126.4 ± 16.3
Diastolic blood pressure (mmHg)	77.8 ± 11.4
Hypertension (%)	29.2
Therapy for hypertension (%)	12.2
Triglycerides (mg/dL)	104 (71, 159)
HDL cholesterol (mg/dL)	56.9 ± 14.8
Dyslipidemia (%)	34.2
CMI	0.93 (0.54, 1.64)
High CMI (%)	22.7
Therapy for dyslipidemia (%)	5.3
Hemoglobin A_1c_ (%)	5.45 ± 0.69
Therapy for diabetes (%)	4.0
Diabetes (%)	6.2
MetS (%)	11.2

Shown are a number, frequencies, means with standard deviations, and medians with interquartile ranges in parentheses.

CMI, cardiometabolic index; HDL, high-density lipoprotein; MetS, metabolic syndrome.

### Odds ratios of the second and third tertile groups versus the first tertile group of hemoglobin or leukocyte count for high CMI and metabolic syndrome

By using logistic regression analysis, odds ratios for cardiometabolic risk, evaluated by high CMI and metabolic syndrome, of the second and third tertiles versus the first tertile for hemoglobin or leukocyte count were estimated (Table 2). In the multivariable analysis, leukocyte count and hemoglobin were added to the explanatory variables in analysis of tertile groups for hemoglobin and leukocyte count, respectively. The odds ratios tended to be higher with an increase in the tertile and were all significantly high when compared with the reference level both in univariable analysis and multivariable analysis, except for the odds ratio of the second versus first tertile groups of hemoglobin for metabolic syndrome in univariable analysis.

**Table 2.** Odds Ratios for High Cardiometabolic Index and Metabolic Syndrome of the Second and Third Tertile Groups of Hemoglobin or Leukocyte Count Versus the Corresponding First Tertile

	High CMI	MetS
Hemoglobin		
Univariable		
First	1.00: Reference	1.00: Reference
Second	1.50 (1.33–1.69)^**^	1.11 (0.95–1.30)
Third	2.28 (2.04–2.55)^**^	1.76 (1.52–2.03)^**^
Multivariable		
First	1.00: Reference	1.00: Reference
Second	1.49 (1.32–1.68)^**^	1.32 (1.11–1.57)^**^
Third	2.10 (1.87–2.36)^**^	2.27 (1.93–2.67)^**^
Leukocyte count		
Univariable		
First	1.00: Reference	1.00: Reference
Second	1.93 (1.71–2.17)^**^	1.67 (1.42–1.95)^**^
Third	2.89 (2.57–3.25)^**^	2.11 (1.81–2.46)^**^
Multivariable		
First	1.00: Reference	1.00: Reference
Second	1.79 (1.58–2.02)^**^	1.74 (1.47–2.06)^**^
Third	2.46 (2.16–2.80)^**^	2.36 (1.98–2.82)^**^

Shown are odds ratios with 95% confidence intervals in parentheses. In multivariable analysis, age, habits of smoking, alcohol drinking and regular exercise, a history of medication therapy for dyslipidemia, and leukocyte count (for analysis of hemoglobin as an explanatory variable) or hemoglobin (for analysis of leukocyte count as an explanatory variable) were used as other explanatory variables. Asterisks denote significant differences from the reference level (^**^P < 0.01).

### Odds ratios for high CMI and metabolic syndrome in nine groups classified by tertiles for hemoglobin and leukocyte count

Odds ratios versus the group consisting of the first tertiles of both hemoglobin and leukocyte count for high CMI (Table 3) and metabolic syndrome (Table 4) were estimated in the other eight groups consisting of the tertiles of hemoglobin and leukocyte count. All of the odds ratios were significantly high when compared with the reference level (1.00) of the group consisting of the first tertiles of hemoglobin and leukocyte count, except for the odds ratios for metabolic syndrome of the group consisting of the second tertile of hemoglobin and the first tertile of leukocyte count. The odds ratios tended to be higher with an increase in each tertile of hemoglobin and leukocyte count. In the multivariable analysis, the odds ratios with 95% confidence intervals of the third (highest) tertiles of both hemoglobin and leukocyte count were 5.30 (4.26–6.59) for high CMI (Table 3) and 4.98 (3.73–6.66) for metabolic syndrome (Table 4). Table 5 shows the regression coefficients and standard errors in each multivariable logistic regression analysis for relationships of hemoglobin and leukocyte count with high CMI and metabolic syndrome. Each regression coefficient was greater than the corresponding estimated standard error, and thus multicollinearity is unlikely to occur. This is due to the high events per variable in our analysis.

**Table 3.** Odds Ratios for High Cardiometabolic Index of Each Subject Group Consisting of Tertiles for Hemoglobin and Leukocyte Count Versus the First Tertile Group of Both Hemoglobin and Leukocyte Count

	Leukocyte count		
	First tertile	Second tertile	Third tertile
Hemoglobin			
First tertile			
Univariable	1.00: Reference	1.86 (1.48–2.33)^**^	2.63 (2.10–3.30)^**^
Multivariable	1.00: Reference	1.84 (1.46–2.32)^**^	2.62 (2.05–3.34)^**^
Second tertile			
Univariable	1.45 (1.14–1.83)^**^	2.83 (2.28–3.50)^**^	3.65 (2.95–4.52)^**^
Multivariable	1.51 (1.19–1.91)^**^	2.89 (2.31–3.60)^**^	3.58 (2.83–4.53)^**^
Third tertile			
Univariable	2.08 (1.65–2.62)^**^	3.55 (2.88–4.36)^**^	5.77 (4.73–7.05)^**^
Multivariable	2.19 (1.72–2.78)^**^	3.59 (2.89–4.45)^**^	5.30 (4.26–6.59)^**^

Shown are odds ratios with 95% confidence intervals in parentheses. In multivariable analysis, age, habits of smoking, alcohol drinking, and regular exercise, and a history of medication therapy for dyslipidemia were used as other explanatory variables. Asterisks denote significant differences from the reference level (^**^P < 0.01).

**Table 4.** Odds Ratios for Metabolic Syndrome of Each Subject Group Consisting of Tertiles for Hemoglobin and Leukocyte Count Versus the First Tertile Group of Both Hemoglobin and Leukocyte Count

	Leukocyte count		
	First tertile	Second tertile	Third tertile
Hemoglobin			
First tertile			
Univariable	1.00: Reference	1.65 (1.25–2.18)^**^	1.67 (1.25–2.22)^**^
Multivariable	1.00: Reference	1.77 (1.30–2.39)^**^	2.65 (1.87–3.75)^**^
Second tertile			
Univariable	1.08 (0.80–1.45)	1.70 (1.29–2.24)^**^	1.92 (1.46–2.53)^**^
Multivariable	1.34 (0.97–1.85)	2.23 (1.65–3.00)^**^	3.02 (2.19–4.17)^**^
Third tertile			
Univariable	1.42 (1.06–1.92)^*^	2.29 (1.78–2.96)^**^	3.35 (2.63–4.27)^**^
Multivariable	2.12 (1.52–2.95)^**^	3.79 (2.82–5.08)^**^	4.98 (3.73–6.66)^**^

Shown are odds ratios with 95% confidence intervals in parentheses. In multivariable analysis, age, habits of smoking, alcohol drinking, and regular exercise, and a history of medication therapy for dyslipidemia were used as other explanatory variables. Asterisks denote significant differences from the reference level (^*^P < 0.05; ^**^P < 0.01).

**Table 5.** Comparison of the Regression Coefficients and Standard Errors in Each Logistic Regression Analysis for Relationships of Hemoglobin and Leukocyte Count with High Cardiometabolic Index and Metabolic Syndrome

	Regression coefficient	Standard error
Second tertile of hemoglobin and first tertile of leukocyte count		
High CMI	0.411	0.122
MetS	0.290	0.165
Third tertile of hemoglobin and first tertile of leukocyte count		
High CMI	0.783	0.123
MetS	0.750	0.170
First tertile of hemoglobin and second tertile of leukocyte count		
High CMI	0.609	0.118
MetS	0.568	0.155
Second tertile of hemoglobin and second tertile of leukocyte count		
High CMI	1.060	0.113
MetS	0.800	0.151
Third tertile of hemoglobin and second tertile of leukocyte count		
High CMI	1.277	0.110
MetS	1.332	0.150
First tertile of hemoglobin and third tertile of leukocyte count		
High CMI	0.962	0.125
MetS	0.973	0.178
Second tertile of hemoglobin and third tertile of leukocyte count		
High CMI	1.276	0.120
MetS	1.105	0.165
Third tertile of hemoglobin and third tertile of leukocyte count		
High CMI	1.667	0.111
MetS	1.606	0.148

Shown are regression coefficients and standard errors in the multivariable logistic regression analysis for high CMI and MetS of each subject group consisting of tertiles of hemoglobin and leukocyte count versus the subject group of the first tertiles of hemoglobin and leukocyte count.

### AUC in ROC analysis for HMI in relation to high CMI and metabolic syndrome in different age groups

Using individual values of hemoglobin concentration and leukocyte count, we propose a new index named HMI (See the Methods section.). AUCs in ROC analysis for HMI and leukocyte count in relation to high CMI and metabolic syndrome were compared in different age groups (Table 6). The AUCs regarding high CMI and metabolic syndrome tended to be smaller with an increase in age, although all of the AUCs were significantly larger when compared with the reference level of 0.500, except for the AUC for relationship between leukocyte count and metabolic syndrome in the age group of 60–65 years. AUCs of the relationships of high CMI and metabolic syndrome with HMI in each age group tended to be larger than those with leukocyte count. The prevalence of metabolic syndrome tended to be higher with an increase in age (30–34 years, 3.1%; 35–39 years, 5.5%; 40–44 years, 7.5%; 45–49 years, 10.9%; 50–54 years, 15.2%; 55–59 years, 17.0%; 60–65 years, 19.7%).

**Table 6.** Area Under the Receiver-Operating Characteristic Curve in Receiver-Operating Characteristic Analysis for Hematometabolic Index and Leukocyte Count in Relation to High Cardiometabolic Index and Metabolic Syndrome in Each Age Group

Age group (number)	AUC (High CM1)	AUC (MetS)
30–34 years (*n* = 723)		
HMI	0.683 (0.635–0.730)^**^	0.785 (0.712–0.858)^**^
Leukocyte count	0.638 (0.613–0.663)^**^	0.661 (0.620–0.702)^**^
35–39 years (*n* = 2031)		
HMI	0.669 (0.640–0.698)^**^	0.693 (0.643–0.743)^**^
Leukocyte count	0.635 (0.615–0.655)^**^	0.619 (0.590–0.648)^**^
40–44 years (*n* = 2009)		
HMI	0.658 (0.631–0.685)^**^	0.679 (0.638–0.720)^**^
Leukocyte count	0.644 (0.617–0.671)^**^	0.641 (0.599–0.683)^**^
45–49 years (*n* = 1704)		
HMI	0.651 (0.621–0.680)^**^	0.635 (0.593–0.677)^**^
Leukocyte count	0.624 (0.595–0.654)^**^	0.598 (0.558–0.639)^**^
50–54 years (*n* = 1600)		
HMI	0.657 (0.628–0.687)^**^	0.625 (0.586–0.664)^**^
Leukocyte count	0.615 (0.585–0.646)^**^	0.584 (0.546–0.623)^**^
55–59 years (*n* = 1766)		
HMI	0.618 (0.586–0.650)^**^	0.599 (0.564–0.635)^**^
Leukocyte count	0.607 (0.575–0.638)^**^	0.597 (0.563–0.631)^**^
60–65 years (*n* = 1084)		
HMI	0.617 (0.577–0.657)^**^	0.576 (0.534–0.618)^**^
Leukocyte count	0.589 (0.549–0.630)^**^	0.539 (0.498–0.580)

Shown are AUCs with 95% confidence intervals in parentheses. Asterisks indicate significantly larger AUCs compared with the reference level (^**^P < 0.01).

AUC, area under the ROC curve; HMI, hematometabolic index; ROC, receiver-operating characteristic.

### ROC analysis for the relationship between HMI and metabolic syndrome in subjects at 30–34 years of age and 30–39 years of age

**FIG. 1.**
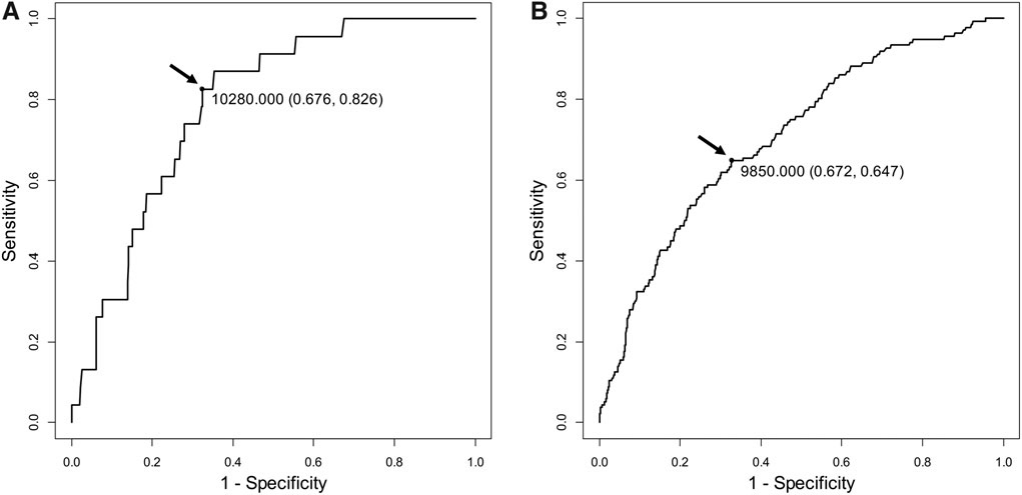
ROC curves for HMI to discriminate metabolic syndrome in subjects 30–34 years of age **(A)** and 30–39 years of age **(B)**. Cutoff points of HMI, yielding maximal sensitivity plus specificity for predicting metabolic syndrome, in the ROC curves were determined. The value, indicated by an *arrow* in each figure, is a cutoff value. The specificity and sensitivity of each cutoff value are also shown in the figures. HMI, hematometabolic index; ROC, receiver-operating characteristic.

The cutoff value of HMI was estimated in ROC analysis for the relationship between HMI and metabolic syndrome in young subjects at 30–34 years of age (Fig. 1A) and 30–39 years of age (Fig. 1B). In the ROC analysis for subjects 30–39 years of age, the AUC was 0.707 (95% confidence interval: 0.663–0.751), and the cutoff of HMI was 9850 with a sensitivity of 64.7% and a specificity of 67.2%. A similar cutoff value of HMI (10,280) with a higher sensitivity (82.6%) was obtained in ROC analysis for subjects at 30–34 years of age.

### Odds ratios of subjects with versus subjects without high HMI for high CMI and metabolic syndrome

Logistic regression analysis for the relationships of high HMI with high CMI and metabolic syndrome was performed using the aforementioned cutoff value of HMI (9850). As shown in Table 7, odds ratios of subjects with versus subjects without high HMI were significantly higher than the reference level in all of the age groups and tended to be lower with an increase in age.

**Table 7.** Crude and Adjusted Odds Ratios for High Cardiometabolic Index and Metabolic Syndrome of Subjects With Versus Subjects Without High Hematometabolic Index in Each Age Group

	High CMI	MetS
Univariable		
30–34 years	3.43 (2.35–5.00)^**^	11.85 (3.49–40.28)^**^
35–39 years	2.64 (2.12–3.30)^**^	3.26 (2.21–4.80)^**^
40–44 years	2.67 (2.16–3.29)^**^	3.06 (2.18–4.28)^**^
45–49 years	2.43 (1.93–3.05)^**^	2.26 (1.66–3.08)^**^
50–54 years	2.57 (2.03–3.25)^**^	2.27 (1.72–3.00)^**^
55–59 years	2.00 (1.56–2.55)^**^	1.81 (1.39–2.37)^**^
60–65 years	1.89 (1.36–2.62)^**^	1.64 (1.17–2.31)^**^
Multivariable		
30–34 years	3.31 (2.24–4.89)^**^	12.77 (3.66–44.53)^**^
35–39 years	2.47 (1.96–3.10)^**^	3.16 (2.10–4.75)^**^
40–44 years	2.69 (2.16–3.35)^**^	3.80 (2.64–5.47)^**^
45–49 years	2.33 (1.84–2.95)^**^	2.35 (1.69–3.26)^**^
50–54 years	2.50 (1.96–3.20)^**^	2.57 (1.88–3.50)^**^
55–59 years	1.92 (1.49–2.48)^**^	2.13 (1.58–2.85)^**^
60–65 years	1.83 (1.30–2.56)^**^	2.15 (1.49–3.11)^**^

Shown are odds ratios with 95% confidence intervals in parentheses. In multivariable analysis, age, habits of smoking, alcohol drinking, and regular exercise, and a history of medication therapy for dyslipidemia were used as other explanatory variables. Asterisks denote significant differences from the reference level (^**^P < 0.01).

## Discussion

Cardiometabolic risk evaluated by prevalences of metabolic syndrome and high CMI was higher in subjects with both high hemoglobin concentration (third tertile) and high leukocyte count (third tertile) in peripheral blood than in subjects with either high hemoglobin or high leukocyte count. Thus, high hemoglobin concentration and high leukocyte count are thought to show synergistic increasing effects on cardiometabolic risk in middle-aged men. This finding led us to propose a new index, HMI, reflecting both hemoglobin concentration and leukocyte count. In the former study on the relationship between polycythemia and cardiometabolic risk,^8^ the tendency of the results of logistic regression analysis for this relationship [crude odds ratios: 2.22 (1.94–2.53) for high CMI and 1.96 (1.66–2.32) for metabolic syndrome; adjusted odds ratios: 2.18 (1.91–2.50) for high CMI and 2.39 (2.02–2.85) for metabolic syndrome] was similar to the tendency of the results in the present study (Table 2), in which tertile groups were used for classification of hemoglobin levels, while two groups with and without polycythemia were used in the former study.

In ROC analysis for the relationship between HMI and metabolic syndrome, moderate accuracy was obtained in young age groups of 30–34 years and 30–39 years. Using the database of subjects 30–39 years of age with a sufficient number of those with metabolic syndrome, we estimated the cutoff value for HMI, which was 9850 with a sensitivity of 64.7% and a specificity of 67.2%. A higher sensitivity (82.6%) with a similar cutoff value (10,280) was obtained in the analysis using the younger subject group (30–34 years). As shown in the results of AUCs in different age groups (Table 6), the associations of HMI with metabolic syndrome and high CMI tended to be weaker with an increase of age. Moreover, the odds ratios for metabolic syndrome and high CMI of subjects with versus subjects without high HMI tended to be lower with an increase of age (Table 7). These findings agree with results of a recent study showing that the association between polycythemia and cardiometabolic risk was weaker in elderly men than in young men.^23^

Although the reason for the age-dependent decline in the association of HMI with cardiometabolic risk is unknown, one possible explanation is that, compared with young individuals, elderly individuals are prone to have larger varieties of confounding factors for the relationship between HMI and cardiometabolic risk, for example, hemopoietic and cardiopulmonary functions. Higher prevalence of cardiometabolic risk in older individuals may also be related to the above age-dependent decline. In fact, the prevalence of metabolic syndrome tended to be higher with an increase in age. Because AUC values tended to be lower (<0.7) after the age of 40 years (Table 6), the cutoff value of HMI was determined by analyzing data for young subjects (30–39 years of age). Thus, further studies are needed to determine whether HMI is useful for evaluating cardiometabolic risk in elderly individuals.

In multivariable logistic regression analysis, the associations of hemoglobin or leukocyte count with high CMI and metabolic syndrome were not changed by adjustment for leukocyte count or hemoglobin concentration (Table 2), suggesting that the association between hemoglobin and cardiometabolic risk was independent of leukocyte count and that the association between leukocyte count and cardiometabolic risk was independent of hemoglobin. These results agree with the above-described findings that hemoglobin concentration and leukocyte count show synergistic effects on cardiometabolic risk. Since leukocyte count is a predictive marker for cardiovascular disease,^24^ HMI was compared with leukocyte count alone by using AUC in ROC analysis, in which high CMI and metabolic syndrome were used as outcomes. AUCs of the relationships of high CMI and metabolic syndrome with HMI in each age group tended to be larger than those with leukocyte count (Table 6). Therefore, HMI is thought to be superior to leukocyte count alone as a discriminator of cardiometabolic risk.

Since both hemoglobin concentration and leukocyte count in peripheral blood are usually included in blood examinations for individuals receiving a general health checkup, HMI will be a useful marker for evaluating the risk of future cardiovascular events, although the formula for calculating HMI may need to be revised to obtain higher accuracy.

There are some limitations of this study. Hemoglobin concentration and leukocyte count in peripheral blood were independently associated with cardiometabolic risk evaluated by CMI and metabolic syndrome, and hemoglobin concentration and leukocyte count were suggested to show synergistic increasing effects on cardiometabolic risk. However, as mentioned above, the association between HMI and cardiometabolic risk tended to be weaker with an increase in age. Thus, it remains to be determined whether HMI is useful for elderly men to discriminate the risk of cardiovascular diseases. The subjects of this study were Japanese male workers, and thus further studies using databases of women and subjects with other ethnicities are needed to confirm the present findings.

HMI is not available for individuals having low hemoglobin concentration (13 g/dL or lower) and/or low leukocyte count (3000/μL or lower) because their HMI values are zero or lower. The outcomes used in this study were high CMI and metabolic syndrome, which consist of plural risk factors for cardiovascular disease and have been shown to be associated with cardiovascular risk.^25–27^ Therefore, future studies using cardiovascular events as outcomes in ROC analysis are needed to confirm the usefulness of HMI. Although it would be interesting to compare HMI and other inflammatory markers, for example, C-reactive protein, fibrinogen, amyloid A, and interleukin 6, regarding the association with cardiometabolic risk, we had no additional measures to discriminate the risk in this study. Since this study is cross-sectional in its design, further prospective studies and clinical trials are needed to confirm the usefulness of HMI as a new index for predicting cardiovascular events.

## Conclusions

High hemoglobin concentration and high leukocyte count were independently associated with high CMI and metabolic syndrome and showed synergistic increasing effects on cardiometabolic risk in middle-aged men. We propose a new index, named HMI, defined as the product of hemoglobin concentration (g/dL)-minus-13.0 and leukocyte count (/μL)-minus-3000. HMI is thought to be a possible marker for discriminating cardiometabolic risk, and this needs to be tested in future studies.
